# *SLC4A4* compound heterozygous mutations in exon–intron boundary regions presenting with severe proximal renal tubular acidosis and extrarenal symptoms coexisting with Turner’s syndrome: a case report

**DOI:** 10.1186/s12881-018-0612-y

**Published:** 2018-06-18

**Authors:** Shoko Horita, Enver Simsek, Tulay Simsek, Nilgun Yildirim, Hiroyuki Ishiura, Motonobu Nakamura, Nobuhiko Satoh, Atsushi Suzuki, Hiroyuki Tsukada, Tomohito Mizuno, George Seki, Shoji Tsuji, Masaomi Nangaku

**Affiliations:** 1 Division of Nephrology and Endocrinology, The University of Tokyo Hospital, 7-3-1, Hongo, Bunkyo, Tokyo, 113 0033 Japan; 2 Department of Paediatric Endocrinology, Eskisehir Osmangazi University School of Medicine, Esogu Meşelik Yerleşkesi, 26480 Eskisehir, Turkey; 3 Department of Ophthalmology, Eskisehir Osmangazi University School of Medicine, Esogu Meşelik Yerleşkesi, 26480 Eskisehir, Turkey; 4 Department of Neurology, The University of Tokyo Hospital, 7-3-1, Hongo, Bunkyo, Tokyo, 113 0033 Japan; 5 Yaizu City Hospital, 1000, Dobara, Yaizu, 425 0055 Japan; 6 Department of Molecular Neurology, The University of Tokyo Hospital, 7-3-1, Hongo, Bunkyo, Tokyo, 113 0033 Japan; 7 Institute of Medical Genomics, International University of Health and Welfare, 4-3, Kozunomori, Narita-shi, Chiba-ken, 286 8686 Japan

**Keywords:** *SLC4A4*, NBCe1, Proximal renal tubular acidosis, Compound heterozygous mutations, mRNA surveillance, Nonsense-mediated decay

## Abstract

**Background:**

Congenital NBCe1A deficiency with the *SLC4A4* mutation causes severe proximal renal tubular acidosis, which often comprises extrarenal symptoms, such as intellectual disability and developmental delay, glaucoma, cataract and band keratopathy. To date, almost all mutations have been found to be homozygous mutations located in exons.

**Case presentation:**

We performed direct nucleotide sequencing analysis of exons and exon–intron boundary regions of the *SLC4A4* in a patient presenting with severe renal proximal tubule acidosis, glaucoma and intellectual disability and her parents without these signs. The examination revealed compound heterozygous mutations in exon–intron boundary regions, c.1076 + 3A > C and c.1772 − 2A > T, neither of which have been reported previously. While the former mutation was found in the mother, the latter was found in the father. The transcript of the *SLC4A4* gene was almost undetectable, and the patient was also diagnosed with Turner’s syndrome.

**Conclusions:**

We identified two novel *SLC4A4* mutations, c.1076 + 3A > C and c.1772 − 2A > T. When presented in a compound heterozygous state, these mutations caused a phenotype of severe renal proximal tubular acidosis along with glaucoma and mental retardation. This is the first report of congenital proximal renal tubular acidosis carrying compound heterozygous *SLC4A4* mutations in exon–intron boundary regions. We suggest that an mRNA surveillance mechanism, nonsense-mediated RNA decay, following aberrant splicing was the reason that the *SLC4A4* transcript was almost undetectable in the proband.

## Background

The electrogenic sodium bicarbonate cotransporter type 1 (NBCe1) is involved in sodium-coupled bicarbonate transport and has three variants: NBCe1A, which is mainly expressed in the kidney proximal tubule; NBCe1B, in the pancreatic ducts and salivary gland and NBCe1C, in the brain astrocytes. NBCe1A plays an essential role in the regulation of acid–base homeostasis by reabsorbing sodium and bicarbonate filtered in the glomeruli. NBCe1A is encoded by a member of the solute carrier family *SLC4A4*. Mutations in *SLC4A4* cause severe proximal renal tubular acidosis (pRTA), with a plasma pH of 7.04–7.27 and a plasma bicarbonate concentration of 3–17 mmol/L. These patients often present with other extrarenal symptoms, such as glaucoma, band keratopathy and growth retardation [1, 2] (OMIM 604278).

To date, 15 mutations of NBCe1A have been found as follows: 13 homozygous (p.Gln29* [3], p.Arg298Ser [1, 4], p.Ser427Leu [5], p.Thr485Ser [6], p.Gly486Arg [7], p.Arg510His [1], p.Trp516* [8], p.Leu522Pro [9], p.Asp721Thrfs*30 [10], p.Leu738del [11], p.Ala799Val [6], p.Arg881Cys [6] and p.Ser982Aspfs*4 [12]), one compound-heterozygous, p.Arg510His and p.Gln913Arg [13], and one 3’-UTR mutation creating an AU-rich element, c.*206G > A [14]. All these mutations exhibit moderate–to–severe pRTA, except c.*206G > A. Moreover, these mutations are located in the coding region, except p.Ser982Aspfs*4, which involves the intron and generates a premature stop codon and c.*206G > A. To date, no symptomatic mutations with the involvement of splice sites have been found for *SLC4A4*.

Here, we report a case of compound heterozygous mutations located in the splicing regions with severe pRTA.

## Case presentation

The patient was a 7-year-old Turkish girl born to non-consanguineous parents. She was being followed up since 3 years of age because of bilateral glaucoma and was prescribed medicines [50 mL of Sholl solution and anti-acidosis capsule (three times a day)] and eye drops (β-blocker and carbonic anhydrase inhibitor). Her mother had oligohydramnios; the patient had intrauterine growth retardation and was born prematurely. There was no family history of any inherited diseases, cataract or pRTA. Both her weight and height were below the 3rd percentile, and she demonstrated intellectual disability. However, the other physical examinations, including neurological signs, were unremarkable.

The laboratory tests were as follows: Na, 139 mmol/L; K, 3.1 mmol/L; Cl, 110 mmol/L; blood urea nitrogen, 12 mg/dL and creatinine, 0.9 mg/dL. Blood gas analysis revealed a pH of 7.22, HCO_3_^−^ concentration of 11 mmol/L and P_CO2_ of 29 mmHg. In addition, urinalysis revealed a pH of 5, no protein and no glucose. The urinary excretion of amino acids was normal, and the urinary β2-microglobulin level was 110 μg/L (normal: < 240 μg/L). These investigations revealed that the patient had pRTA without Fanconi syndrome – generalized dysfunction of proximal tubule. Considering her short stature, the levels of thyroid hormones, IGF-I and IGFBP3 were normal. Furthermore, whereas the renal ultrasound revealed a 9-mm diameter parenchymal stone in the right kidney, brain MR imaging revealed no intracranial calcification. Neither of the parent showed these symptoms.

At the age of 9, our patient presented with micrognathia, fish-mouth, epicanthal folds, ptosis, low-set ears, a short neck with a low hairline, a broad shield-like chest, wide-spaced nipples, hypoplastic areolae, cubitus valgus and short fourth metacarpals, with other symptoms due to NBCe1A absence such as dental abnormalities, suggesting the coincidence of Turner’s syndrome. In addition, her weight and height were still below the 3rd percentile. Hormonal investigation data were as follows: FSH, 69.3 mIU/mL (normal: 4.5–20.0 mIU/mL); LH, 15.9 mIU/mL (3.5–14.0 mIU/mL) and oestradiol, < 5.0 pg/mL, suggesting hypergonadotropic hypogonadism. Her karyotype was 45, XO which confirmed the diagnosis of Turner’s syndrome.

### Genetic analysis

From 200 μL of peripheral blood samples obtained from the patient and her parents, we extracted DNA using the QIAamp DNA Blood Mini Kit (Qiagen Inc.) according to the manufacturer’s instructions. Similarly, we extracted RNA from 1 to 2 mL of the peripheral blood sample using the Isogen (Nippon Gene) or the QIAamp RNA Blood Mini Kit (Qiagen Inc.) according to the manufacturer’s instructions. Then, the complementary DNA (cDNA) of the patient was synthesised from the polyA(+) RNA of the peripheral white blood cells using the cDNA Synthesis Kit (Takara) as previously described [15] or the RevertAid First Strand cDNA Synthesis Kit (Thermo Scientific) according to the manufacturer’s instructions.

The polymerase chain reaction (PCR) condition used was as follows: denaturation for 9 min at 95 °C, followed by 35 cycles of 95 °C for 1 min, 60 °C for 1 min and 72 °C for 1 min, with a final extension at 72 °C for 7 min. PCRs were performed using a thermal cycler PerkinElmer GeneAmp PCR System 2400 (PerkinElmer Japan, Applied Biosystems Division, Tokyo, Japan). The DNA sequence of each PCR product was determined using the Sanger sequencing method, with the primers shown in Table 1, in an ABI3100 sequence analyser (Life Technologies, Carlsbad, CA). In addition, AmpliTaq™ (Roche) and attached buffers were used for PCR. The primers in Table 1 were used for the analyses of exons and splicing site sequences of *SLC4A4*.

**Table 1:** Primers used for genome PCR

Exons	Forward	Reverse
Exon 1	CTGCGAGGGCATGAGCTTTAG	CCAACATCATGCCCATTG
Exon 2	GGAAGTGCTGGAAGGGGTG	CCAGAGGAAGATGTTATGGAAG
Exon 3	CATATCTGTGTACCCTGTGTC	GTCACCGTGGCATTAGCAG
Exon 4	CTCTTCAGAAGAATCCTAGTG	GTTGTCTGCACGTAAAGGTC
Exon 5	GTGGCTAGCTAGAACATGTTGC	GACAGTATAAAAGTCAAACAGTC
Exon 6	GGTGAATTCTAGACCTAACC	CAAATGACCGTACCTCATGC
Exon 7	GGACTATCAGAGCATGGCTGG	AAACATCGCCAAAGCATGTC
Exon 8	GTTAGATAGCAGAAAGAAATAAC	CCCCATAAAACCATCACCAC
Exon 9	GTTTCATCGTAAGTGGTTAAG	CAGCAGCAGGCCAGAAGCAAAG
Exon 10	GACTTTGTTCTTCATTCTTG	CTCACATCTGAACATTCCAG
Exon 11	CTGGCTAAAGTAGAGTTTCAC	CCTTGCAAATCCCACAGTTT
Exon 12	CATTGTGCCCTTATGTTGTTATTA	GTTACGTATGTGTTCATGCC
Exon 13	GTTTCACCCTCCAGTGCT	TTTTCCTTTCAGCACATTCAGA
Exon 14	GATACCTCCTTCAATTTGTTG	TCAGGAGGATGATAGTTACAATACG
Exon 15	CTTCATTCTCTAGCTCATAACTG	CTGGTTCTGCGGACTCTTAAG
Exon 16	CTCTTTCAAGGAGTTTAACTTAC	ATCACTGAAACCTCTGATG
Exon 17	GTTTATACGCTATCCTTGAG	CTGCTTCAGTGTGTTACAGAAC
Exon 18	GCATACTAGTTAGAGGTCACTAAG	GCAGGTGAATGGTGAAGTAG
Exon 19	GACCATTCCTTTGTCCTCTG	CTGATCAAAGTGATGAGGTC
Exon 20	CAAGATCAGGTCTGTCATACTC	GAGTAATACACCACATGTCCAG
Exon 21	TGAGGGGGAAAGAAGGAATGC	AGCCATTGGAAAAACTGGGGA
Exon 22	CTAGAGTCTTAGCTTAATACCTTG	GAGACGAAGGAGAACAAGAAG

The sequences of primers used for the detection of β-actin and fragments of *SLC4A4* coding sequences were as follows: hACTB748F, 5’-ATTGGCAATGAGCGGTTC-3′, and hACTB979R, 5’-TCTTCATTGTGCTGGGTGC-3′; exon2-3bridgeF, 5’-GTTGGTGGAGATGATTGTTGAC-3′, and exon6-7bridgeR, 5’-GTCATGGAACACCTCATCAGAC-3′; exon5-6bridgeF, 5’-TGCCCACAAGGTTCTTGTTC-3′, and exon8-9bridgeR, 5’-ACCACAGAACCGTCCAGTTC-3′.

The quantitative RT-PCR (qRT-PCR) was performed according to its instructional manual, with TaqMan Gene Expression Master Mix (Applied Biosystems, Foster City, CA, USA), TaqMan Gene Expression Assays (Hs00186798_m1 for *SLC4A4*, Hs01060665_g1 for β-actin; all from Applied Biosystems) and sequence detection system (7500 Fast Real-time PCR System; Applied Biosystems). The expression level was quantified relative to the abundance of β-actin cDNA.

### Identification of *SLC4A4* gene mutations

**Fig. 1:**
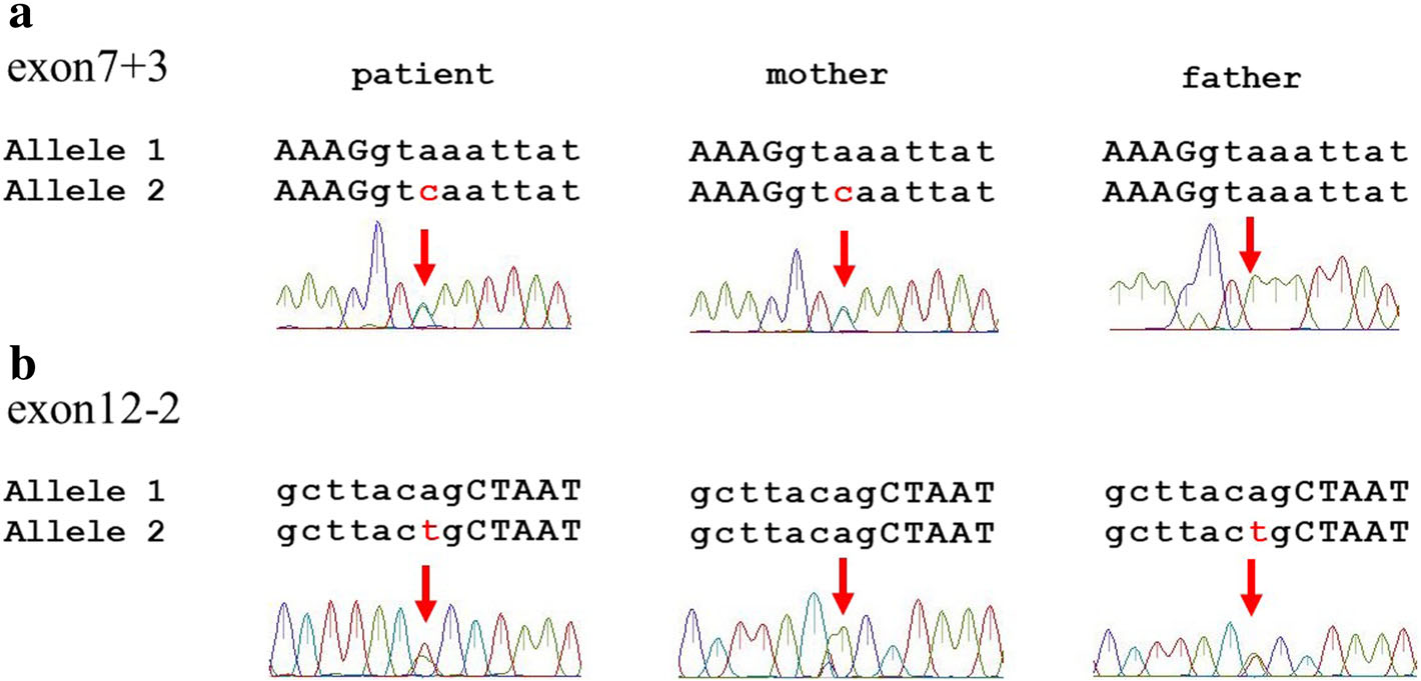
Identification of two novel *SLC4A4* mutations. The sequence analysis for the proband and the parents revealed the presence of compound heterozygous mutations c.1076 + 3A > C (**a**) and c.1772 − 2A > T (**b**)

The sequencing analysis of the *SLC4A4* gene (OMIM 603345, ENST00000340595.3, NM_003759.3) across each exon, including the adjacent intronic sequences of approximately 100 base pairs of the proband, revealed two heterozygous mutations as follows: (a) c.1076 + 3A > C, three bases after the end of exon 7 (Fig. 1a and b) c.1772 − 2A > T, two bases before the beginning of exon 12 (Fig. 1b). In addition, we analysed the *SLC4A4* genes of her parents and confirmed that her mother and father had heterozygous mutations c.1076 + 3A > and c.1772 − 2A > T, respectively. No other mutations in the *SLC4A4* gene were detected in the genomes of the patient or her parents. Of note, both mutations are absent from the ExAC database (http://exac.broadinstitute.org/).

### *In silico* analysis

Owing to the locations of both mutations on the splice sites, we performed *in silico* assays to elucidate whether the splicing sites were altered in the proband.

We used the webtools ‘Splice Site Score Calculation’ (http://rulai.cshl.edu/new_alt_exon_db2/HTML/score.html) [16, 17], ‘NetGene2 Server’ (http://www.cbs.dtu.dk/services/NetGene2/), ‘Human Splicing Finder Version 3.1’ (http://www.umd.be/HSF3/index.html) and ‘Berkeley Drosophila Genome Project Splice Site Prediction by Neural Network’ (http://www.fruitfly.org/seq_tools/splice.html) for the *in silico* evaluation of these mutations. The ‘Splice Site Score Calculation’ demonstrated that the scores of the original sequences were 9.2 and 9.8, whereas the scores of the aberrant sequences were 2.5 and − 1.2, respectively (in order of c.1076 + 3A > C, c.1772 − 2A > T). Because the mean score of the 3′ splice site in constitutive exons was 7.9 and that of the 5′ splice site in constitutive exons was 8.1, the proband’s data suggested that the mutations could cause aberrant splicing (data not shown).

In contrast, ‘NetGene 2 Server’ suggested that there may be no splice donor site for the c.1076 + 3A > C mutation and that there may be an aberrant acceptor splice site in c.1772 − 2A > T (data not shown), whereas The ‘Human Splicing Finder Version 3.1’ suggested that in c.1772 − 2A > T the acceptor splice site is broken (data not shown). The ‘Berkeley Drosophila Genome Project Splice Site Prediction by Neural Network’ [18] suggested that c.1076 + 3A > C mutation abolishes the original splice donor site and provides an alternative splice donor site (c.1076 + 197_198GT). It also suggested that c.1772 − 2A > T abolishes the original acceptor site and provides alternative acceptor sites (c.1772 − 29_ − 28AG, c.1772 − 37_ − 36AG, c.1772 − 168_ − 167AG).

**Fig. 2:**
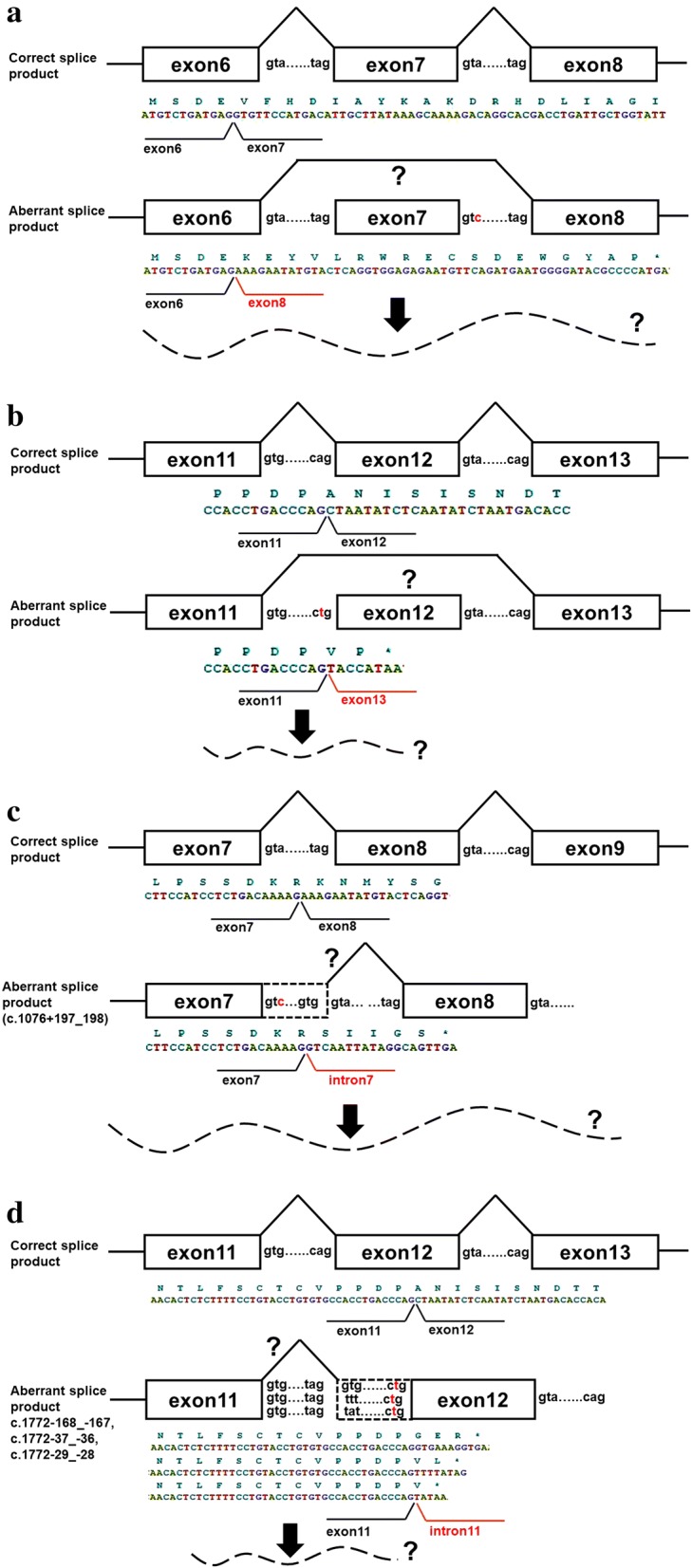
Models of aberrant transcription caused by splice site mutations. Models of exon skipping by c.1076 + 3A > C mutation (**a**) and c.1772-2A > T mutation (**b**) are shown. Alternatively, models of intron retention and activation of cryptic splice sites by c.1076 + 3A > C mutation (**c**) and c.1772-2A > T mutation (**d**) are shown. Cryptic splice sites are predicted using Splice Site Prediction by Neural Network in Berkeley Drosophila Genome Project. All the four models predict premature stop codons

Furthermore, the estimated models of aberrant transcription according to previous literature [19, 20] suggested the appearance of nonsense codons in each allele of the patient’s genome (Fig. 2a and b).

### The analysis of the patient’s cDNA

**Fig. 3:**
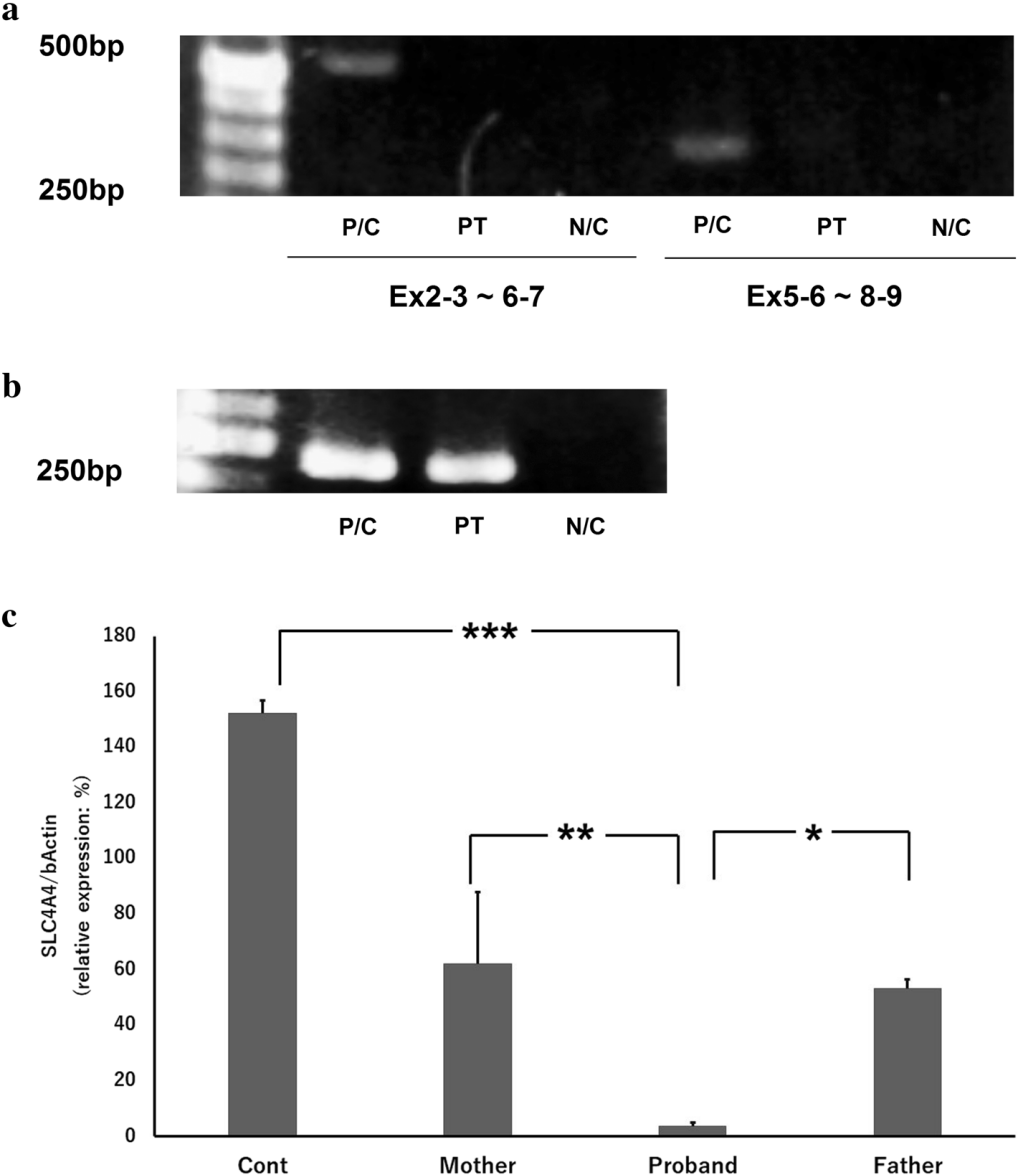
Identification of fragments of *SLC4A4* coding sequences (**a**) and β-actin (as a control; **b**) in cDNA of the proband and control healthy human. Reverse-transcription PCR (RT-PCR) products of the *SLC4A4* coding region were not detected in the proband. In contrast, the fragment of β-actin was detected in the proband’s cDNA. PT, proband; P/C, positive control (the cDNA from white blood cells of a healthy human as a template); N/C, negative control (no template). **c** quantitative RT-PCR (qRT-PCR) in cDNA provided by the proband, parents and control. *n* = 2 each. ***: *p* < 0.01, **: *P* < 0.05, *: *P* < 0.1

We tried to assess the sequence of the *SLC4A4* cDNA because we obtained the cDNA of the proband from her mRNA. However, we did not detect the expression of *SLC4A4* (Fig. 3a). Then, owing to the detection of the expression of β-actin in the proband’s cDNA (Fig. 3b), the absence of the *SLC4A4* cDNA sequence suggested that *SLC4A4* was either not expressed or expressed at extremely low levels in the proband.

We further tried to confirm the expression amount of the *SLC4A4* compared to β-actin in the patient by the qRT-PCR. The relative expression ratio of *SLC4A4* to β-actin in the proband was extremely low compared to that of the healthy human control (*P* < 0.01), mother (*P* < 0.05) and father (*P* < 0.1) (Fig. 3c).

## Discussion and conclusions

To the best of our knowledge, this is the first report of pRTA caused by *SLC4A4* mutation with unique aberrant sequences and compound heterozygous mutations that are both splice site mutations.

Typical patients with NBCe1A deficiency present with severe pRTA accompanied by extrarenal symptoms, such as ocular symptoms – band keratopathy, cataract and glaucoma, growth and mental retardation and sometimes intracranial calcification [4]. This patient also had severe pRTA (HCO_3_^−^ concentration: 11 mmol/L), glaucoma and growth and mental retardation, significantly coinciding with typical pRTA phenotypes because of NBCe1A deficiency.

All cases of previously determined pRTA because of the NBCe1A mutation had mutations involving exons; some mutations showed intracellular retention (p.Arg298Ser, p.Arg510His, p.Arg881Cys and p.982Serfs*4) or no protein expression (p.Gln29* and p.Trp516*) in polarised MDCK cells. Functional analyses of p.Arg298Ser, p.Thr485Ser, p.Gly486Arg, p.Arg510His, p.Leu522Pro, p.Asn721Thrfs*30, p.Ala799Val, p.Arg881Cys and p.982Serfs*4 elucidated the pathophysiology of NBCe1A deficiency, the acid–base regulation of the proximal tubule and the functional roles of NBCe1A [6, 7, 10, 12, 21]. When expressed in *Xenopus* oocytes or HEK293 cells, the function of this mutant NBCe1A was reduced (p.Arg298Ser, p.Thr485Ser, p.Gly486Arg, p.Arg510His and p.Arg881Cys) or almost abolished (p.Leu522Pro, p.Asn721Thrfs*30 and p.Ala799Val), as determined by electrophysiological analysis and/or cell pH measurement.

In our patient, reverse-transcription PCR (RT-PCR) revealed that *SLC4A4* mRNA was not detected in the peripheral leukocytes, although the primers were designed well upstream of the mutations. Moreover, qRT-PCR showed that the expression ratio of *SLC4A4* to β-actin was extremely low in the patient. Hence, we could not identify the exact sequence of the aberrantly spliced *SLC4A4* mRNA in the patient. Because we could detect the expression of the house-keeping gene, β-actin in the patient’s cDNA, the most probable speculation would be that the expression level of *SLC4A4* mRNA was at extremely low levels or almost zero in this patient.

Perhaps, the extremely low expression of *SLC4A4* in the qRT-PCR and abundance below detection limit of *SLC4A4* mRNA in the RT-PCR could be attributed to some type of mRNA surveillance, a mechanism that controls the quality of mRNA in eukaryotes [22, 23]. Three types of mRNA surveillance found are nonsense-mediated mRNA decay (NMD), nonstop decay (NSD) and no-go decay (NGD) [24–27]. NMD was first identified in mRNA surveillance and is widely known to accelerate the degradation of aberrant mRNAs having a premature terminate codon. While NSD is a mechanism that degrades mRNAs lacking a stop codon, NGD eliminates mRNAs with elongation stalls. Mutations in the borderline splicing regions may cause exon skipping, the activation of cryptic splice sites and the creation of a pseudo-exon within an intron and/or intron retention [28].

Figure 2a and b show the estimated models of exon skipping, because previous literatures indicated that mutations in splice donor sites [19, 29, 30] and splice acceptor sites [31, 32] can cause exon skipping. Figure 2a shows a model that an A > C change at position + 3 of the splice donor site of intron 7 causes skipping of exon 7, which results in a premature stop codon. Similarly, Fig. 2b shows a model that an A > T change at position − 2 of the splice acceptor site upstream of exon 12 causes skipping of exon 12, which results in a premature stop codon [20, 31, 32].

Other possible consequences of the splice site mutations would be intron retention and activation of cryptic splice sites. The all cryptic splice sites suggested by ‘Berkeley Drosophila Genome Project Splice Site Prediction by Neural Network’, a splice donor site (c.1076 + 197_198 corresponding to GU at the 5′ end of the intron) generated by c.1076 + 3A > C, and splice acceptor sites (c.1772 − 29_ − 28, c.1772 − 37_ − 36, and c.1772 − 168_ − 167 corresponding to AG at the 3′ end of the intron) generated by c.1772 − 2A > T, are predicted to cause premature stop codons (Fig. 2c and d) when activated. These aberrantly spliced mRNAs through exon skipping or activation of cryptic splice sites may have been degraded by NMD because of premature stop codons.

Hence, we speculate the occurrence of NMD in our patient, resulting in the absence of NBCe1A and the pathogenesis of pRTA. Because *SLC4A4* cDNAs were detected in the peripheral leukocytes of patients with most of previously reported mutations [1, 3, 5–13, 21], this study revealed a novel mechanism in congenital NBCe1A deficiency.

Turner’s syndrome is one of the most common genetic disorders, resulting from the complete or partial deletion of one X chromosome [33, 34]. The clinical features of Turner’s syndrome usually comprise symptoms such as short stature, abnormalities as mentioned earlier, ovarian insufficiency, and cardiac complications. While the reason for the delayed diagnosis of Turner’s syndrome remains unclear, this delay occurs very often, as mentioned in previous studies [33]. In our patient, the growth retardation was first attributed to congenital pRTA, which may have delayed the diagnosis of Turner’s syndrome. The total clinical timeline is summarized in Table 2.

**Table 2:** Medical History Timeline

Year	Clinical findings	Diagnosis
2011-		Bilateral glaucoma was found; Sholl solution and anti-acidosis capsule was prescribed
2015	Physical Examination: Weight and height, below the 3rd percentile; Mental retardation. Other physical examinations, neurological signs: unremarkable. Serum: Na, 139 mEq/L; K, 3.1 mEq/L; Cl, 110 mEq/L; blood urea nitrogen, 12 mg/dL; creatinine, 0.9 mg/dL, pH, 7.22; HCO_3_^−^, 11 mmol/L; P_CO2,_ 29 mmHg. Free T4, TSH, IGF-I, IGFBP3; normal. Urinalysis: pH, 5.0; no protein; no glucose. Urinary excretion of amino acids, normal; β2-microglobulin, 110 μg/L (normal: < 240 μg/L). Renal ultrasound, a 9-mm diameter parenchymal stone in the right kidney. Brain MR imaging, no intracranial calcification.	Diagnosed as proximal renal tubular acidosis Identification of *slc4a4* mutation
2017	Weight, 21 kg (<3rd percentile); height, 116 cm (<3rd percentile). Physical examination: micrognathia, “fish-mouth” appearance, dental abnormalities, epicantal folds, ptosis, low-set ears, short neck with low hairline, broad shield-like chest, wide-spaced nipples, hypoplastic areolaes, cubitus valgus, short fourth metacarpals. Hormonal investigations: FSH, 69.3 IU/L (normal: 4.5–20.0 mIU/L); LH, 15.9 IU/L (normal: 3.5–14.0 mIU/L) and estradiol, < 5.0 pg/mL. Karyotype, 45. Obstetric ultrasound; uterine and gonadal hypoplasia.	Diagnosed as Turner’s syndrome

In conclusion, we identified novel compound heterozygous splice site mutations of *SLC4A4* in a patient presenting typical pRTA. In our opinion, NBCe1A deficiency caused by NMD, a type of mRNA surveillance mechanisms, could be held accountable for these symptoms.

## Abbreviations

NMD: Nonsense-mediated decay
PCR: Polymerase chain reaction
pRTA: Proximal renal tubular acidosis
cDNA: Complementary DNA
NSD: Nonstop decay
NGD: No-go decay
